# Noncoding RNAs in Extracellular Fluids as Cancer Biomarkers: The New Frontier of Liquid Biopsies

**DOI:** 10.3390/cancers11081170

**Published:** 2019-08-14

**Authors:** Barbara Pardini, Alexandru Anton Sabo, Giovanni Birolo, George Adrian Calin

**Affiliations:** 1 Department of Experimental Therapeutics, The University of Texas MD Anderson Cancer Center, Houston, TX 77030, USA; 2 Department of Medical Sciences, University of Turin, 10124 Turin, Italy; 3 Unit of Molecular Epidemiology and Exposome, Italian Institute for Genomic Medicine (IIGM), 10126 Turin, Italy; 4 Department of Pediatrics, Marie Curie Emergency Clinical Hospital for Children, 077120 Bucharest, Romania; 5 Center for RNA Interference and Non-Coding RNAs, The University of Texas MD Anderson Cancer Center, Houston, TX 77030, USA; 6 Department of Leukemia, The University of Texas MD Anderson Cancer Center, Houston, TX 77030, USA

**Keywords:** noncoding RNAs, body fluids, liquid biopsy, long noncoding RNAs, microRNAs, piRNAs

## Abstract

The last two decades of cancer research have been devoted in two directions: (1) understanding the mechanism of carcinogenesis for an effective treatment, and (2) improving cancer prevention and screening for early detection of the disease. This last aspect has been developed, especially for certain types of cancers, thanks also to the introduction of new concepts such as liquid biopsies and precision medicine. In this context, there is a growing interest in the application of alternative and noninvasive methodologies to search for cancer biomarkers. The new frontiers of the research lead to a search for RNA molecules circulating in body fluids. Searching for biomarkers in extracellular body fluids represents a better option for patients because they are easier to access, less painful, and potentially more economical. Moreover, the possibility for these types of samples to be taken repeatedly, allows a better monitoring of the disease progression or treatment efficacy for a better intervention and dynamic treatment of the patient, which is the fundamental basis of personalized medicine. RNA molecules, freely circulating in body fluids or packed in microvesicles, have all the characteristics of the ideal biomarkers owing to their high stability under storage and handling conditions and being able to be sampled several times for monitoring. Moreover, as demonstrated for many cancers, their plasma/serum levels mirror those in the primary tumor. There are a large variety of RNA species noncoding for proteins that could be used as cancer biomarkers in liquid biopsies. Among them, the most studied are microRNAs, but recently the attention of the researcher has been also directed towards Piwi-interacting RNAs, circular RNAs, and other small noncoding RNAs. Another class of RNA species, the long noncoding RNAs, is larger than microRNAs and represents a very versatile and promising group of molecules which, apart from their use as biomarkers, have also a possible therapeutic role. In this review, we will give an overview of the most common noncoding RNA species detectable in extracellular fluids and will provide an update concerning the situation of the research on these molecules as cancer biomarkers.

## 1. Introduction

In oncology, a biomarker is defined as a measurable alteration in a sample that is associated with cancer condition or that could have a predictive or prognostic value. In the era of precision medicine, high sensitivity and specificity are essential characteristics for a good biomarker, especially if it is meant to be used for detecting early cancer stages [1]. Highly predictive biomarkers are not only useful for screening and for a better diagnosis, but mostly they could serve as a starting point to understand the biological pathways and regulatory mechanisms involved in the pathology and could serve as a potential target for drugs [2].

**Figure 1:**
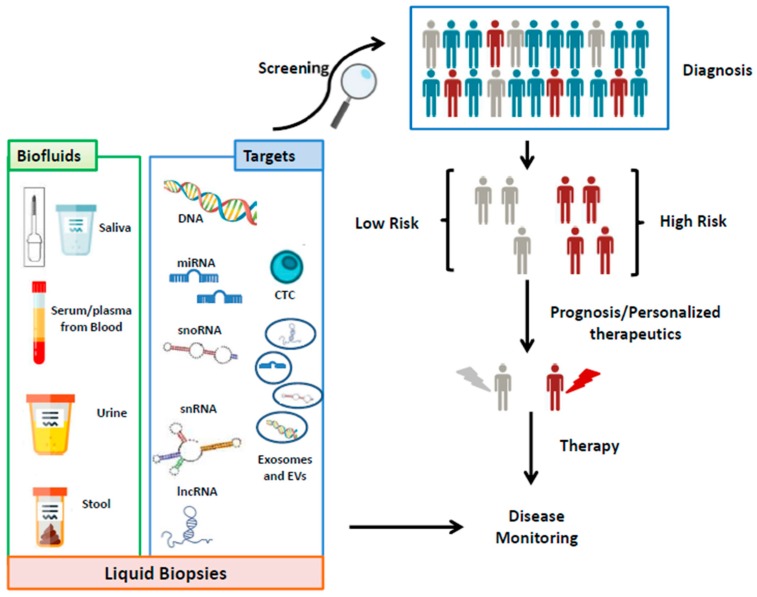
An effective management of cancer diagnosis screening by using body fluids and molecular biomarkers (CTC: circulating tumor cells; snoRNA: small nucleolar RNA; snRNA: small nuclear RNA; lncRNA: long noncoding RNA; EVs: extracellular vesicles).

An effective management of cancer could be reached with effective cancer screening programs, an early diagnosis, an efficient classification of the prognosis, but also with a good plan for monitoring disease progression and efficacy of the therapy [3]. Besides the recent advances in the molecular biology, the histological examination of biopsy tissues remains the gold standard for the majority of cancer diagnoses. However, biopsies are unpleasant and risky for the patients, as well as expensive and time-consuming. In addition, they are difficult to perform and require a certain expertise of the pathologists [4]. Consequently, there is a large interest in the application of alternative methodologies to search for noninvasive cancer biomarkers. Noninvasive biomarkers, and in particular circulating nucleic acids, represent a better option for patients because they are easier to be sampled, less painful, and potentially more economical. When comparing solid with liquid biopsies, a series of other advantages of the latter must be mentioned. Firstly, liquid biopsies can be used in the early detection of cancer, before radiologic and imaging events have detected the presence of a tumor (which can be assessed by traditional biopsy). Secondly, especially when considering histologically heterogeneous tumors, liquid biopsies may better characterize the tumor as a whole, while classic biopsies, analyzing only specific fragments, might miss some of the molecular characteristics. Thirdly, the possibility with liquid biopsies of repeated sampling permits a better and more dynamic disease progression monitoring and modulation of treatment. Liquid biopsies can be potentially applied in tracking tumor clonal evolution over time, testing for tumor resistance mechanisms, assessing treatment response, and detecting minimal residual disease, elements which constitute the fundamental basis of personalized medicine. Lastly, in the patient follow-up, liquid biopsies can be used as a convenient tool in the early detection of recurrence (Figure 1) [5,6].

Biomolecules such as proteins, DNA, and RNA are potentially optimal candidates as biomarkers, and can be isolated from several biological materials. In the last years, great attention has been dedicated to the detection of such biomarkers in body fluids [7]. Among these molecules of interest, RNA is most probably the best candidate. In fact, compared with protein biomarkers, RNA detection presents higher sensitivity and specificity, and the analyses are less expensive. On the other hand, compared with DNA, RNA biomarkers provide better dynamic insights of cell regulation and states. Moreover, some specific RNAs (such as circular RNAs (circRNAs) and microRNAs (miRNAs)) are stable in the majority of body fluids (Figure 1) [2].

However, not all of the aforementioned advantages of liquid biopsies are available for RNA-based analyses. For example, the tracking of clonal events through liquid biopsies is rather possible through circulating DNA (ctDNA) or circulating tumor cells (CTC), while cell-free RNA (cfRNA) cannot be used for this purpose [5]. Moreover, since these molecules are a reflection of biological processes throughout the body, other concurrent biological events (e.g., sepsis) are known to majorly influence the circulating RNA expression profiles [8] and may interfere with the oncologic theranostic process. Even so, under these circumstances, RNA-based analytics have non-neglectable advantages and therapeutic applications, which will be further described in this review.

Mammalian genomes are highly transcribed, and the majority of the transcripts do not code for proteins [9,10]. Once excluded messenger RNAs (mRNAs), the remainder of the transcriptome contains vast classes of noncoding RNAs (ncRNAs) that are functional elements not comprehensively studied yet. The cellular repertoire of ncRNAs includes: miRNAs, circRNAs, small housekeeping RNAs such as ribosomal (rRNAs) and transfer RNAs (tRNAs), and long noncoding RNAs (lncRNAs). miRNAs, the most studied small ncRNAs, account for less than 2% of the total ncRNAs [9]; much remains to be discovered for the remaining noncoding transcriptome [11].

The majority of ncRNAs have regulatory functions: they could be transcribed constantly together with coding RNAs, or they could be the results of environmental stimulation as a response to certain specific situations [12]. Interestingly, hypoxia has been shown to induce the transcription of genetic regions that are not transcribed in nonhypoxic settings, generating the hypoxia-induced noncoding ultraconserved transcripts, also called HINCUTs, which are implicated in the oncogenic process [13]. Most probably, not all the ncRNAs are functional; however, most of them, other than miRNAs could be essential for both physiological function and development, as well as playing a fundamental role in disease such as cancer [10,14].

With the development and affirmation of next-generation sequencing (NGS) technology, the identification and quantification of various types of RNAs have become much easier and more powerful. For example, with NGS it has been possible to detect a large amount of new Piwi-interacting RNAs (piRNAs) or discover new genomic elements such as Pyknons (“peak-non-s”), a class of short DNA sequence motifs that can be also contained in lncRNAs [15]. NGS enables, in fact, not only to detect novel transcripts but also to detect with great accuracy nucleic acids in samples like plasma, urine, and other body fluids and extracellular matrices in which the amount of such species is very low [16]. These technological advances have revolutionized ncRNAs discovery.

In the present review, we focused on the emerging potential of ncRNAs as cancer biomarkers in liquid biopsies.

## 2. The Circulating Transcriptome

cfRNAs were discovered in 1999 with the description of Epstein–Barr virus RNA in the plasma of nasopharyngeal carcinoma patients [17]. This was followed by the discovery of free-circulating mRNAs in melanoma patients’ serum [18]. With few exceptions, the large majority of cfRNAs are degraded by RNAse activity in blood [19,20]. However, a great improvement in the field has been reached with the discovery of cell-free miRNAs in the blood of cancer patients [21,22]. In contrast to long molecules of RNA species (i.e., mRNA), circulating miRNAs are highly stable: they are resistant to degradation at room temperature for up to 4 days and in deleterious conditions such as boiling, stable after multiple freeze–thaw cycles, and insensitive to high or low pH [22]. The high potentialities of miRNAs as cancer biomarkers have generated an increased interest in these molecules. Finally, the advent of more powerful and available NGS technologies (RNA-seq) has enlarged the interest also to other ncRNAs that could be present in body fluids (for example lncRNAs and other small ncRNAs), either as free-circulating molecules or packed into microvesicles such as exosomes [23] (Figure 1).

Considering technological advances from recent years, heterogeneity in pre-analytical and analytical steps is still present across published studies. This heterogeneity largely affects the reported results. For example, there are several available techniques for exosome purification (ultracentrifugation, separation by specific solutions like Exoquick, etc.), and isolating specifically tumor-derived exosomes would be desirable, although practical and technical hindrances still exist. Likewise, nucleic acids isolation through different commercially available kits, quantification methods, and a number of methodologies for expression levels assessment (ranging from microarray and RT-qPCR to NGS) all account for heterogeneity and pose technological challenges to researchers. For more information on technical aspect, pitfalls, and limitations please refer to Anfossi et al. [14]. Also, for an up-to-date analysis on the extracellular vesicles please check Xu et al. [24].

Besides the increased application to body fluids analyses of RNA-seq in the last years, most available publications are focused on miRNAs, while the research on cell-free ncRNAs other than miRNAs is a recent field of study. Huang and colleagues presented one of the first works on the whole circulating transcriptome, performed by RNA-seq on plasma extracellular vesicles from healthy subjects [25] and cancer patients [26]. The majority of the obtained reads were from miRNAs (about 40%), but there was also a good representation of rRNAs, lncRNAs, and piRNAs. The presence of a multitude of ncRNAs species in various body fluids able to characterize them, has been observed in various studies [27,28,29,30].

Few studies investigated the cell-free whole circulating transcriptome in search of cancer biomarkers [31,32,33,34] and, in such cases, the proportion of ncRNA species was variable depending on the body fluids of origin but also on the nucleic acids detection method. The origin of cfRNAs is still an issue for the scientific community. Extracellular RNAs can be released in a passive way as a derivate of the cell activity and death [35], or can be actively secreted from cells, either associated with membrane-derived extracellular vesicles (such as exosomes and microparticles) [22,36] or alternatively conjugated with proteins (lipoproteins, Argonaute 1 and 2 etc.) [37].

Exosomes secreted from a malignant cell often reflect the molecular composition of the cell of origin [38]. The loading of molecules into the exosomes is a selective process not completely clarified yet. In the specific case of cancer and tumor-reactive immune cells, exosomes and other microvesicles are released into the bloodstream or other body fluids to transfer specific paracrine or endocrine messages [23], to regulate and induce specific tumor functions such as growth, migration, anti-cancer drug resistance, and cell cycle control [39]. Yu et al. found that exosomes from highly metastatic pancreatic cancer cells contained differentially expressed proteins involved in exosome-mediated intercellular communication [40]. Tumor-derived exosomes carry specific molecular signatures that characterize different stages of tumor progression, with an interesting perspective to use these molecules as diagnostic and prognostic markers [41] or even as therapeutic targets [42,43,44].

## 3. MiRNAs in Biological Fluids

miRNAs are endogenous small ncRNA molecules of 19–22 nucleotides of length that post-transcriptionally modulate the expression of protein-coding genes through binding to specific sequences on target genes [45]. miRNAs pair with their target mRNAs with imperfect binding, therefore they can regulate hundreds of transcripts, at least potentially [46]. miRNAs have a tissue-specific pattern of expression and often are expressed in an aberrant way in many diseases, including cancer [47,48,49,50]. Interestingly, it has been shown in murine models that miRNA genes are located in genetic loci associated with cancer susceptibility [51]. miRNAs have been detected in various extracellular body fluids such as plasma and serum [37,52,53], cerebrospinal fluid (CSF) [54], saliva [55], breast milk [56], urine [57], and ovarian follicular fluid [58].

The extracellular miRNAs can be delivered to target cells as free-circulating molecules (often associated with proteins, especially Argonaute 2) or loaded into vesicles such as exosomes, microvesicles, and apoptotic bodies and behave with a hormone-like mechanism for intercellular communication, acting as autocrine, paracrine, and/or endocrine regulators to modulate cellular activities [23,59,60]. In fact, they can regulate the activity of host cells, but, more interestingly, precursors and mature miRNAs in microvesicles could also be secreted and transferred to recipient cells where they are functionally active [61,62,63]. Therefore, in the last years, hormone-like activities of long-distance circulating miRNAs have been recognized [23,64].

In Table 1, we report a selection of the most recent studies reporting miRNAs circulating in body fluids that have been detected as possible cancer biomarkers. The list may not be complete, as a large number of papers published in the last years investigated the role of miRNAs as biomarkers for specific cancer diagnosis. The studies listed here are the most recent available, but we invite readers to search for more details about specific cancers in more cancer-type-focused reviews [65].

The majority of the studies available were investigating serum, plasma, or vesicles derived from these specimens [25,66,67,68,69,70,71,72,73,74,75,76,77,78,79,80,81,82,83,84,85,86,87,88,89,90,91,92], but there are also some examples of miRNAs found altered in urine [57,66,93,94,95,96], saliva [66,97], cerebrospinal fluid [91], and stool [66] (Table 1). One of the first described miRNA, and one of the most recurrently detected as a cancer biomarker is miR-21, implicated in a wide range of cancers ranging from the digestive to respiratory tract, hematological, gynecological, and brain malignancies. miR-21 is an oncomiR implicated in several signaling pathways, its upregulation resulting in inactivation of several tumor suppressors [98].

Being very abundant in serum and plasma samples, the let-7 family has been detected by multiple researchers in several types of cancers, such as hepatocellular cancer (HCC) [66], gastric [76], colorectal [66], but also urologic—urinary bladder [57], prostate [25,84,85,86,89,95,99], pulmonary [66], breast [90], and ovary [66,92,100]. Likewise, miR-155 has been also reported as dysregulated in serum/plasma samples in gastroenterology malignancies (esophageal [66], pancreatic [73], and colorectal [80] cancers), pulmonary [67], breast [101], ovary [102], and additionally, hematologic malignancies (acute leukemia [66] and lymphoma [66]). Another miRNA detected in serum and plasma exosomes and found to be dysregulated in several cancers is miR-144 [25,86,87].

Many miRNAs have been reported to be dysregulated and involved in several types of cancers, which makes sense when thinking of their general action as either oncogenes or tumor suppressors. Still, in the quest of identifying precise disease-specific biomarkers, the question of miRNA specificity has to be addressed. We compared those miRNAs reported as biomarkers in body fluids (Table 1) with the miRcancer database (https://mircancer.ecu.edu) [103,104,105], that lists miRNAs dysregulated in cancer, mainly from tumor tissue samples. We reported which miRNAs were found to be dysregulated either in body fluids, tumor tissues, or both (B, T, and BT, respectively) in Supplementary Table S1A–C. The cross-matched table was generated in order to have an overview of the miRNA “specificity” in certain tumors or specimens. Few of the reported miRNAs were really specific to one single type of cancer. miR-21-5p, a known oncomiR, was the one implicated in the largest number (19) of tumors in both body fluids and tissues; miR-101-3p was the most frequently altered in tissues, only within 20 types of cancer, and miR-197-3p was the most implicated in body fluids, only within 5 cancer types. We found only a couple of miRNAs that were specific to a single cancer category (Table S1A,B). As an example, miR-127-3p was associated only with gynaecologic cancers (breast, ovarian and cervical) in body fluids (but also to other cancers in tissues). On the other hand, miR-1285-3p was associated only to urologic cancers: prostatic cancer in body fluids, and renal cell carcinoma in tissues (Table 1 and Table S1A–C). This observation is explainable by the fact that miRNAs are implicated in general oncogenic pathways (e.g., miR-21 and let-7 are implicated in the Ras and NF-kB pathways [106]), and to some extent also in more specific pathways (e.g., the implication of miR-155 in the Diffuse large B-cell lymphoma (DLBCL) genesis through interaction with SHIP1 and C/EBPβ pathways [107,108]).

On the other hand, some miRNAs were specific to just one cancer type. For instance, 55 individual miRNAs were dysregulated only in one type of cancer in both body fluids and tissues, 214 in tissues and 239 in body fluids (Table S1B).

The highest number of miRNAs dysregulated in both body fluids and tumor tissues were in breast cancer, HCC, and chronic leukemia with respectively 51, 299, and 85 miRNAs in common (Table S1A,C). Some miRNAs resulted altered in a large number of cancers. As an example, miR-21-5p was a good biomarker in tissues and body fluids for leukemia, lymphoma, pancreatic, gastric, esophageal, colorectal, renal, breast, prostate, and ovarian cancers, but also for non-small-cell lung cancer (NSCLC) and glioma. miR-17-5p, another known oncomiR, was altered in leukemia, lymphoma, esophageal, liver, pancreatic, gastric, colorectal, and breast cancer, as well as glioma and NSCLC (Table S1B,C).

Under these circumstances, understanding the implication of miRNAs in specific pathways and finding the most sensitive and specific ones is still challenging. Many studies have not focused on single miRNAs, but proposed specific cancer diagnostic panels and diagnostic trees with greater specificity and sensitivity compared with single miRNA. For example, we proposed a 3-miRNAs panel for bladder cancer diagnosis [57], while regarding brain cancers, several researchers described multiple miRNA panels useful in the glioma diagnosis [109,110,111], as recently reported by us [91]. This trend also complies to the pragmatic needs of clinical medicine, where accurate, standardized tools are needed, not only for the diagnostic, but also for the prognostic and therapeutic management.

The number of studies covering miRNAs implicated in all cancers and detectable as biomarkers in body fluids is extremely high, also considering the increase of interest of researchers on these molecules in the last years. For a complete in-depth review investigating all the single studies of free-circulating and exosome miRNAs as cancer biomarkers, please refer to [23,66,67,100,112,113,114,115,116,117,118,119,120,121,122]. For example, the role of exosomal miRNAs in body fluids for prostate cancer diagnosis has been investigated by many groups and recently reviewed by Valentino et al. [123] and Fabris et al. [124]. Likewise, the measurement of miRNAs derived from blood and cerebrospinal fluid in brain tumors has been recently covered by Petrescu et al. [91]. For an extensive review of miRNAs involved in gastric cancer refer to Bhat et al. [76]; regarding ovarian cancer, refer to the work of Giannopoulou et al. [92], while several reviews are available on breast cancer, focusing on the complex milieu of miRNAs in liquid biopsies [90,125,126,127]. Regarding haematologic malignancies, a recent review by Drokow et al. [128] summarized the main miRNAs with potential use as diagnostic biomarkers. Concerning the general implication of miRNAs in acute myeloid leukemia (AML), please refer to Liu et al. [129]. Likewise, the complex implications of miRNAs in chronic lymphocytic leukemia (CLL) are reviewed by Mirzaei et al. [130] and Ciccone et al. [131], while for chronic myeloid leukemia (CML), see Litwińska et al. [132]. Finally, for more information about pediatric hematologic malignancies, please refer to Carvahlo de Oliveira et al. [133].

## 4. PiRNAs in Biological Fluids

piRNAs are a class of 26–31 nucleotide small ncRNAs that is transcribed from genome intergenic regions with a Dicer-dependent mechanism [251,252]. They are the largest known class of small ncRNAs [253,254,255] and work in transposon silencing and in specific transcriptional regulation functions. For example, it has been demonstrated their role in embryonic development and spermatogenesis [256]. piRNAs are 5-monophosphated and 2′-O-methyl, modified at the 3′ terminal, which are typical features of stable molecules [257]. They have a tissue-specific expression profile, and their abundance in cancer allowed researchers to hypothesize their involvement in cancer regulatory processes [251,258]. In one of the last estimates, the presence of different piRNAs encoded in mammalian genomes has been calculated, reaching up to tens of millions [259].

piRNAs have been found either in isolated exosomes or as free-circulating in serum, plasma, saliva, and stool [27,260]. Alterations in circulating piRNA expression levels may work as good cancer biomarkers, with higher sensitivity and specificity than circulating miRNAs [157]. Only a few studies investigated the role of piRNAs as cancer biomarkers in body fluids, and we report a selection of them in Table 1. The large majority of the reported studies were focused on piR-651 and piR-823, but the field is in continuous development and update.

High circulating piRNA levels have been found in gastric [157], colorectal [6,22,261], prostatic [26], and renal [172] cancers, but also in Hodgkin’s Lymphoma [33]. piR-823, for example, was found up-regulated in serum and urine of renal carcinoma patients when compared with healthy controls [172]. piR-651 expression levels in serum were lower in Hodgkin’s lymphoma patients than in healthy controls [192]. Finally, a set of piRNAs, altered in plasma-derived extracellular vesicles could characterize colorectal (piR-019825), prostate (piR-016658 and piR-020496), and pancreatic (piR-001311 and piR-016658) [26] cancers. Interestingly, 31 free-circulating piRNAs in serum were recently found to be significantly dysregulated in colorectal cancer (CRC) patients compared with healthy donors. The levels of piR-5937 and piR-28876, in particular, were able to differentiate between cancer patients and healthy donors with high sensitivity and specificity, and after chemotherapy treatment, the expression levels were again comparable between cases and controls. Vychytilova-Faltejskova et al. concluded that piRNAs could serve as promising noninvasive biomarkers for early detection of colon cancer [159].

## 5. SnRNAs and SnoRNAs

Small nuclear (snRNA) and small nucleolar (snoRNA) RNAs are components of the small ncRNAs transcriptome. Both are 60–300 nucleotides long and transcribed from intronic sequences of coding genes [262,263].

snRNAs are located in the cell nucleus and are fundamental in the RNA–RNA remodeling, in spliceosome assembly, being implicated in the translation process [262]. This class of molecules named “U(n)”, based on their high Uridyl content (e.g., U1, U2–U12), form the spliceosome complex together with specific associated proteins (snRNPs). The spliceosome specifically binds to the pre-mRNA and precisely cleaves intron sequences, aiding the formation of a mature mRNA. During the formation of the spliceosome, snRNAs are implicated in complex RNA–RNA and RNA–protein interactions, which assure the correct binding to the pre-mRNA. Alterations in snRNA structure have been reported, and their functional consequences could be implied in the oncogenic process [264].

Table 1 reports the snRNAs that have been studied in liquid biopsies as cancer biomarkers. The best-known snRNA released into body fluids is U2: altered expression levels of fragments of RNU2 (RNU2-1f) have been detected in serum or plasma of lung, colorectal, pancreatic, and ovarian cancers [156,163,164,175]. Another potentially interesting snRNA that could serve as useful circulating biomarker for breast cancer is U6, which is overexpressed in breast cancer patients, independently of estrogen receptors (ER) status [174].

snoRNAs are hosted in introns of coding and noncoding transcripts [265,266] and are implicated in the post-transcriptional modification of rRNA [267,268]. In recent years snoRNAs have been also recognized as involved in the formation of small nucleolar ribonucleoprotein particles (snoRNP) [262]. Additionally, there is some evidence that snoRNAs can act in miRNA-like post-transcriptional gene silencing [269]. Recently, the existence of cleavage products (20 to 30 base pair, similar in length to miRNAs) from 5′ or 3′ ends of snoRNAs have been described: these species have been termed sdRNA—sno-derived RNAs. The functional oncogenic implication of these products has been investigated in the cancer genome atlas (TCGA) samples, revealing a high sensitivity in distinguishing cancer types on the basis of sdRNA expression profiles. More than their possible use as biomarkers, major implications of these molecules in the oncogenic process, tumor immune milieu, patient prognostic, response to therapy, and aid in targeted therapy have been hypothesized and have a promising outlook, warranting future investigation [270,271].

Table 1 reports the snoRNAs that have been studied in liquid biopsies as cancer biomarkers. Regarding them, the available studies performed in liquid biopsies are mainly on lung cancer. SnoRD33, snoRD66, and snoRD76 were found up-regulated in plasma from NSCLC patients [161], while snoRD33, snoRD66, snoRD42, and snoRD78 were investigated in sputum samples of patients with the same type of cancer [162,272]. Recently, two snoRNAs (snoRA74A and snoRA25) detected in serum exosomes resulted as good biomarkers, able to distinguish pancreatic cancer patients from controls [155].

## 6. LncRNAs

lncRNAs constitute a heterogeneous group of noncoding transcripts of >200 nucleotides that may be distributed in various cellular compartments. They can be intragenic (intronic or antisense) or intergenic [273]. Some of them are stable and highly conserved, while others have a high turnover and are poorly conserved. The biological functions ascribed to this class of ncRNAs are various: from coordinating gene regulation at DNA/RNA level to regulation of protein biogenesis and function [273]. lncRNAs resulted importantly also in chromatin remodeling, as well as transcriptional and post-transcriptional control, interacting with RNA, DNA, and proteins as scaffolds, decoys, and enhancer RNAs. lncRNAs play an important role in carcinogenesis and metastasis, and they seem to be key players in cancer biology [274,275,276]. They are considered potential cancer diagnostic and prognostic factors [277], especially considering that lncRNAs are rather stable and can be detected in body fluids as circulating biomarkers for disease diagnosis [278,279]. Similarly to small ncRNAs, also lncRNAs can be carried through exosomes or other extracellular vesicles to disseminate signals, with the aim to change or regulate recipient cells either locally or at distance. Theoretically, the function of lncRNAs loaded into extracellular vesicles can work in a hormone-like fashion similarly to miRNAs, as we recently argued [23]. The number of investigations on circulating lncRNAs as cancer biomarkers has grown in the last 5–8 years. The most relevant works on this field are reported in Table 2. The majority of the studies conducted on body fluids have been performed on serum/plasma [280,281,282,283,284,285,286,287,288,289,290,291], but only some researchers focused on extracellular- [292,293,294] and urine-derived lncRNAs [295,296,297,298,299], while some others focused on saliva [300,301], bile [302] and gastric juice [303].

In a recent work by Kamel et al., a significant down-regulation of growth-arrest-specific transcript 5 (*GAS5*) expression and up-regulation of SOX2 overlapping transcript (*SOX2OT*) have been observed in NSCLC patients when compared with controls [288].

Wang et al. recently published an interesting work focused on establishing the expression levels of colon cancer-associated transcript 2 (*CCAT2*) in CRC tissues, in serum and in exosomes derived from serum. The authors found that the levels of expression of this lncRNA were significantly over-expressed in CRC tumor tissues, and that this over-expression was reflected in serum and serum-derived exosomes of CRC patients when compared with healthy controls [293]. This data points out the large interest in *CCAT2* that was observed in the last years. Indeed, the overexpression of this lncRNAs in transgenic mice led to spontaneous myeloid malignancies, while patients with myelodysplastic and myeloproliferative malignancies had high bone marrow and circulating levels of this lncRNA [304]. Interestingly, *CCAT2* has been shown to be implicated in cancer metabolism by influencing the glutaminase splicing [305]. For more information regarding the implications of *CCAT1* and *CCAT2* in human cancers, please refer to [306].

Another research group investigated the role of small nucleolar RNA host gene 18 (*SNHG18*) in HCC and found it downregulated in cancer tissues compared with paired adjacent noncancerous tissues. Interestingly, the down-regulation was also confirmed in plasma samples [286]. Other researchers found six aberrantly expressed lncRNAs in HCC tissues with respect to the corresponding normal tissues; however, only small nucleolar RNA host gene 1 (*SNHG1*) expression in cancer tissues correlated with the expression levels measured in plasma from the same patients. Interestingly, plasma *SNHG1* expression levels correlated also with tumor size, TNM staging, and α-fetoprotein levels, demonstrating its potential of being a reliable biomarker for the diagnosis of this cancer [290].

Recent reviews summarized findings regarding the implication of cell-free or exosome-derived lncRNAs [421,422,423] in several types of cancer, while specific reviews focused only on one type of cancer—nasopharyngeal [424], NSCLC [425], prostate [426,427,428], gastric [429], ovarian [430], bladder [431], and multiple myeloma [432]. In a recent meta-analysis on 28 studies, Dai et al. showed that circulating lncRNAs are more accurate markers for NSCLC compared with tissue samples, having an overall high diagnostic efficacy in the distinction of lung cancer [433]. Similar high diagnostic accuracy of lncRNAs was observed by Chen and collaborators in a metanalysis on circulating lncRNAs and HCC [434]. Regarding ovarian cancer, a systematic review based on tissue lncRNAs and survival has been done by Ning et al. [435]. However, to the best of our knowledge, none of the analyzed molecules have been investigated in serum samples. Finally, altered expression levels of *FAM83H-AS1* were found in ovarian cancer tissue samples and proposed as potential circulating biomarkers [436].

A few studies proposed joint panels combining several ncRNAs for better accuracy in cancer diagnosis [173,369]. For example, Eissa et al. proposed a urine lncRNA, *miR-497-HG*, together with miR-324-5p, miR-4738-3p, and mRNA *RCAN1 FOSB* for bladder cancer diagnosis [173].

Notably, since 2012, urine determination of *PCA3* (PROGENSA^®^) is the only FDA-approved lncRNA used in the diagnostic process for prostate cancer [437,438,439], and, as it can be observed in the reported studies, the analysis of miRNAs and lncRNAs in urine-derived exosomes will continue to provide a sensitive resource in diagnosing malignancies of the bladder and prostate, with further promising application in the clinical field [57,295,296,297,298,299,401].

## 7. CircRNAs

Circular RNAs (circRNAs) are a class of lncRNAs characterized by the covalent linkage of their 5′ and 3′ moieties, which gives them a specific circular form [440] and makes them biologically stable and resistant to RNases [441]. They originate from intronic, exonic, intergenic regions, or exonic–intronic junctions and are formed by alternative mRNA backsplicing [442,443]. Although firstly reported in 1979 in human cells [444], for years they were considered splicing error by-products. However, in the last years, with the advent of NGS, they have been reconsidered, and their role as biomarkers rediscovered [445].

The major known biological function of circRNAs is related to gene expression regulation. They act in fact as sponges by competitively binding miRNAs. In this way, they can function as miRNA reservoirs, aid in miRNA transportation, or suppress miRNA binding to target genes [445]. circRNAs can also bind to RNA-binding proteins (RBPs), acting as protein sponges [446]. Moreover, by binding two or more proteins they can act as scaffolds, facilitating enzyme–substrate interaction [445]. As a consequence, circRNAs directly modulate gene expression at the transcriptional and post-transcriptional levels by interacting with elements of the spliceosome and by influencing the balance between canonical and alternative splicing. Therefore, there is a sort of passive competition between circRNAs and their relative linear mRNAs, generating a perturbation in the balance between transcripts, promoting aberrant expression of oncogenes and tumor suppressor genes [443,445].

The interaction between circRNAs, miRNAs, and RBPs is complex and not yet fully understood, posing difficulties in conceptualizing their dysregulation in pathologic situations [443]. However, circRNAs have been investigated in a wide range of diseases [447], including several types of cancer. The majority of the studies on circRNAs associated with cancer focused on tissue biopsies and cell lines, with a wide range of data reported (as reviewed by [448]). There are a few studies that searched for circRNAs only in body fluids [309,356,408,409,418]. The majority of works first detected circRNAs dysregulated in cancer tissues, and then tested if the same signature was reflected also in body fluids [311,328,329,333,352,353,354,355,357,358,359,370,371,398,419,449,450,451]. A recent systematic review on circRNAs included 77 studies and several types of cancers and emphasized the potential role as diagnostic biomarker of single and combined circRNAs with altered expression in plasma and serum [452]. All the circRNAs proposed as cancer diagnostic biomarkers in body fluids are reported in Table 2.

Nine circRNAs analyzed in plasma were found dysregulated in gastric cancer from several research groups (hsa_circ_0001017, hsa_circ_0061276 [353], hsa_circ_0000745 [354], hsa_circ_0000520 [355], hsa_circ_0000190 [357], hsa_circ_0001649 [352], *circKIAA1244* [356], hsa_circ_0000467 [358], and hsa_circ_0000181 [359]. In particular, the first five circRNAs reported above were also recently confirmed by Jiang et al. in a metanalysis as dysregulated in plasma of gastric cancer patients [453].

In another study, the *circMAN1A2* upregulation in serum was associated with several malignancies, with the highest diagnostic accuracy reported for nasopharyngeal carcinoma [309]. Interestingly, in the same type of cancer, also circRNA_0000285 resulted dysregulated in serum and has been reported as good diagnostic and prognostic marker for this tumor [311].

For CRC, two studies found a dysregulation of hsa_circ_0001649 [370] in serum and *circVAPA* [371] in plasma. For bladder cancer, *circPRMT5* up-regulation in tissues was found also in serum and urine exosomes from the same patients [398]. For NSCLC, hsa_circ_0013958 [449], *circFARSA* [450], and hsa_circ_0005962 [451] were reported to be up-regulated in patients’ plasma, while hsa_circ_0086414 [451] resulted down-regulated. Only one study investigated in plasma the expression levels of circRNAs in breast cancer, and hsa_circ_0001785 resulted the only specimen with altered expression in this body fluid [408]. On the other hand, hsa_circ_0109046 and hsa_circ_0002577 were reported as dysregulated in serum extracellular vesicles from endometrial cancer patients [409]. Other interesting results emerged for other circRNAs dysregulated in other cancers: hsa_circ_0001445 [328] and *circSMARCA5* [329] in plasma from HCC patients; serum exosome hsa_circ_007293, hsa_circ_031752, and hsacirc_020135 in papillary thyroid carcinoma [418]; and serum hsa_circ_0000885 in osteosarcoma [419]. Interestingly, the up-regulation of *circLDLRAD3* was reported as a putative diagnostic biomarker for pancreatic cancer [333].

Although circRNAs are very stable, and availability in various body fluids has been reported [454], their study as diagnostic biomarkers in liquid biopsies is mainly based on blood-derived specimens. Only few studies investigated circRNA profiles in other body fluids—saliva [260,308,455], gastric juice [456], and urine [398]. To our knowledge, no cancer circRNA profiling has been reported in feces or CSF.

For more information on the implications of circRNAs in cancer, and a critical approach regarding their use as biomarkers, we recommend referring to Dragomir and Calin [443] and Ragan et al. [445].

## 8. Final Remarks and Future Perspectives

Recent years brought notable advances in the research of ncRNA implications in the oncologic field. A plethora of studies focused on miRNAs, to investigate their involvement in oncogenesis and their potential use as diagnostic and prognostic markers, but also as therapeutic means. This enormous enthusiasm in miRNA investigation was not replicated to the same extent in the investigation of other ncRNAs. Some studies focused on the analysis of lncRNAs as biomarkers, while recent studies revealed new functions and implication of piRNAs in oncogenesis [457]. To date, the scientific community is trying to decipher and understand the complex pathways and interactions by which ncRNAs are implicated in oncogenesis. Many aspects regarding their role and importance need still to be clarified. For instance, it is not known the exact number of ncRNAs species in the human genome. For lncRNAs, it has been estimated a number that ranges from about 5000 to 53,000 species [458]; for miRNAs, there are at least 2000 known species [459], while it has been estimated about 50,000 piRNAs in the mammalian genome [460].

One of the most valuable uses of ncRNAs is as biomarkers for disease. Initially, studies focused on analyzing expression profiles in cancerous tissues. As a reaction, it was expected to find similar dysregulations also in body fluids, as a reflection of the presence of the disease in a surrogate tissue. However, not all findings were replicated in body fluids. To increase the specificity, researchers focused on isolating RNA from circulating exosomes, which indeed revealed more promising results, being an alternative mode of communication between neighboring and distant cells. In this paper, we reported also an overview of the signatures found in body fluids that are reflecting tumor tissue profiles, and their potential use in clinical medicine. Tumor-derived exosomes can originate and reach different cell types also to distant sites, influencing the biological activities of tumors, such as proliferation, invasion and metastasis, immunoregulation, generation of a premetastatic niche, and stimulation of angiogenesis [461]. Recent outcomes indicate that cancer-connected exosomes exhibit specific characteristics in educating macrophages, alter dysbiosis and exacerbate progression of CRC, implicating a role of the host defense system in cancer progression. At present, we are still far from clearly understanding the relationship between exosomes and macrophages and the recognition process; moreover, it is not yet clear how they can interact and influence the microbiome [462]. However, their functions are evidently affected by their cargo, which includes many RNA species including miRNAs and other ncRNAs.

Interestingly, ncRNAs have a potential application also as therapeutics in the near future. The replacement of the tumor suppressor miR-34a, by using a synthetic product, MRX34, was the first miRNA replacement therapy in a phase I clinical trial in humans, which initially reported promising results [463]. Since then, constant advances in using ncRNAs in cancer therapy are being made [464]. Besides being reported to be implicated in predicting chemo- and radioresistance, miRNAs and lncRNAs have been proposed as therapeutic targets, and miRNAs are being used in clinical trials [465]. For example, targeting miR-155 and TP53 in lung cancer improved therapeutic response in vitro [466]. Likewise, siRNA-mediated inhibition of EphA2, potentiated by miR-520d-3p replacement, acts as a tumor suppressor, both in vitro and in vivo [467]. Moreover, targeting miR-29b and miR-125 by suppressing Podoplanin, could aid in blocking invasiveness in glioblastoma [468]. Interactions between miRNAs and Toll-like receptors have been reported, underlining the implication of ncRNAs in cancer immunology [469]. Also, miRNA expression patterns are analyzed in order to better understand and direct immune checkpoint inhibitor therapy [470,471]. For more details on the application of ncRNAs as therapeutics, we recommend referring to [465,472].

With the development of NGS techniques, many researchers have embarked on genome/transcriptome sequencing from a wide set of samples. Due to this specific technology, new species of previously not described ncRNAs have been reported, including for example piRNAs, which have been discovered just in 2006 [457]. The advancements in this field may conduct to the discovery of new, more specific and sensitive molecules to be used as biomarkers and give new insights into the so-called “junk” DNA/RNA.

Although advances in the field are promising, we cannot help from noticing drawbacks in the NGS analyses process and in the research of biomarkers in body fluids. It must be noted that it is not uncommon to find studies focusing on the same type of cancer and analyzing the same body fluid that report contradictory results regarding ncRNA expression profiles, sometimes even in opposite directions of dysregulation. This phenomenon could be explained by the lack of uniformity in study designs, including differences in study group size, population study ethnicity [473], the inclusion or not of cancer risk factors such as smoking status [474], and gender [475]. All these features could influence study outcomes. Moreover, there is an enormous heterogeneity regarding technical steps towards the final results: RNA isolation procedure, exosome separation, NGS library preparation, sequencing, and bioinformatics/biostatistics approaches in analyzing and interpreting results. It is also difficult to establish the more suitable body fluids to be used for biomarker discovery in different types of cancers. Taking for instance CRC, stool would seem an ideal candidate to analyze, being the elected surrogate specimen; however, new studies are emerging on the analysis of sputum or saliva for the detection of biomarkers for CRC [476]. Other issues affecting the reproducibility of the data are directly connected with NGS. For RNA-seq the methodology of preparation of the libraries could be still very variable, and for the moment there is no uniformity in the pipeline of analysis.

Big improvements are now ongoing, and in the near future, we believe many of the drawbacks will be overcome with a better understanding of the ncRNAs’ role as cancer biomarkers.

## Figures and Tables

**Table 1:** Small noncoding RNAs (microRNAs, piwi-interacting RNAs, and sn/snoRNAs) with possible application as diagnostic biomarkers in body fluids.

Cat.	Cancer Type	miRNA	piRNA	sn/snoRNA
**Digestive **	Oral cancer	miR-125a, miR-200a (SA) [97] miR-16, miR-29a (S) [66] miR-125b (P) [66]		
	Nasopharyngeal cancer	miR-548q, miR-630, miR940 (P) [134] miR-16, miR-21, miR-24, miR-155, miR-378 (P) [135], miR-548q, miR-483-5p (P) [136], miR-24 (P) [137] miR-17, miR-20a, miR-29c, miR-223 (S) [138], miR-486-5p, miR-45, miR-100 (S) [139] miR-188-5p, miR-1908, miR-3196, miR-3935, miR-4284, miR-4433-5p, miR-4665-3p, miR-513b (B) [140] miR-31-5p (B) [141] miR-34c-3p, miR-18a-5p (TEP) [142]		
	Esophageal cancer	miR-16, miR-17, miR-20a, miR-30a, miR-155, miR-375 (P) [66] miR-10, miR-18a (S) [66] miR-21, miR-1246 (SE) [67] miR-30a (SE) [67]		
	Hepatocellular carcinoma	let-7b, let-7f, miR-16, miR-17, miR-30e, miR-125a, miR-221, miR-375 (S) [66] miR-18a, miR-221, miR-222, miR-224 (SE) [68] miR-106b (P, SE) [66]		
	Cholangiocarcinoma	miR-29, miR-122, miR-155, miR-192 (S) [143] miR-483-5p, miR-194 (S) [144] miR-150-5p (S) [145] miR-26a (S) [146] miR-106a (S) [147] miR-1281, miR-126, miR-26a, miR-30b, miR-122 (S) [148] miR-21, miR-221 (P) [149] miR-150 [150] (P) miR-412, miR-640, miR-1537, miR-3189 (BI) [148] miR-9, miR-145 * (BI) [151] *Pancreato-biliary cancers* vs. *Healthy Controls:* miR-6075, miR-4294^, miR-6880-5p, miR-6799-5p, miR-125a-3p, miR-4530^, miR-6836-3p, miR-4476 ^ (S) [152] *^ = reported, but no sufficient available data (www.miRbase.org)*		RNU2-1f (BI) [153]
	Gallbladder carcinoma	let-7a, miR-21, miR-187, miR-143, miR-202, and miR-335 (P) [154]		
	Pancreatic cancer	miR-486-5p, miR-938 (P) [69] miR-486-5p, miR-126-3p, miR-106b-3p (P) [70] miR-223 (P) [71] miR-182 (P) [72] miR-10b, miR-30c, miR-106b, miR-155, miR-212 (P) [73] miR-1290 (S) [74] miR-192 (S) [75] miR-21, miR-17-5p (S) [67] miR-210 (P, U) [66]	piR-016658, piR-001311 (PV) [26]	SNORA74A, SNORA25 (S/SE) [155] RNU2-1f (P/S) [156]
	Gastric cancer	miR-199-3p, miR-20a, miR-106b, miR-221, miR-486, miR-451, miR-17-5p, miR-106a, miR-106b, miR-21, let-7a, miR-195-5p (P) [76] miR-21, miR-221, miR-376c, miR-744, miR-378, miR-195-5p, let-7a, miR-196a, miR-451, miR-486 (S) [76] let-7c, let-7i, let-7f, miR-17, miR-17, miR-106b (S) [66] miR-200c (P, S), miR-16 (P, S) [66] let-7a, miR-18a, miR-19b, miR-106a, miR-17, miR-106a/b, miR-106b (P) [66]	piR-823, piR-651 (B) [157]	
	Colorectal cancer (CRC)	miR-25-3p [77] (SE) miR-27a, miR-130a (SE) [78] miR-17, miR-18a, miR-18b, miR-19a, miR-19b, miR-20a, miR-20b, miR-106a (P, SE) [79] miR-21, miR-23a, miR-224, miR-92a, miR-155 (SE) [80] miR-196b-5p (SE) [81] miR-217 [158] (SE) miR-182-5p, miR-149, miR-96-5p (SE) [82] miR-19a, miR-29b, miR-29c, miR-125b, miR-155 (S) [66] miR-92a, miR-17/-92, miR-200c, miR-141, miR-375 (P) [66] let-7f, miR-18a, miR-20a (F) [66] miR-29a (P, S) [66] miR-221 (P, F) [66] miR-106a (F, P) [66] let-7a (SE, P) [66]	piR-019825 (PV) [26] piR-5937; piR-28876 (S) [159] piR-001311, piR-004153, piR-017723, piR-017724, piR-020365 (S) [160]	RNU2-1f (P/S) [156]
**Respiratory**	NSCLC	miR-193b, miR-301, miR-141, miR-200b (S) [83] miR-17-3p, miR-21, miR-106a, miR-146, miR-155, miR-191, miR-192, miR-203, miR-205, miR-210, miR-212, miR-214 (P) [67] miR-34a (B); let-7i, miR-10, miR-19a, miR-19b, miR-29c, miR-30c, miR-34c, miR-125a, miR-125b, miR-200c, miR-141, miR-429 (S); let-7a/b (P) [66] miR-20a (P) [66] miR-30a (P) [66] miR-375 (P) [66] let-7a/b (S/P) [66] let-7f (PV) [66] miR-125b (S, P) [66] miR-155 (S, S/P) [66]		snoRD33, snoRD66, snoRD76 (P) [161] snoRD33; snoRD66; snoRD42; snoRD78 (SP) [162] RNU2-1f (S) [163,164]
	Laryngeal cancer	miR-21, miR-155, miR-221 (P) [165] miR-155 (P) [166] miR-331-3p, miR-603, miR-1303, miR-660-5p, 212-3p (P) [167] miR-31, miR-141, miR-149a, miR-182, let-7a, miR-4853p, miR-122, miR-33, miR-145, miR-223, miR-133a (S) [168] let-7 (S) [169] miRNA-378 (S) [170] miR-221 (S) [171]		
**Urologic **	Renal cell carcinoma	miR-210 (S) [66] miR-221 (P) [66]	piR-823 (S, U) [172]	
	Bladder cancer	miR-375 (UE) [93] miR-21-5p (UE) [94] miR-30a-5p, let-7c-5p and miR-486-5p (U) [57] miR-19a (P); miR-106b (U); miR-210 (S, U) [66] miR-324-5p, miR-4738-3p (together with *miR-497-HG* and *RCAN1 FOSB* mRNA) (U) [173]		
	Prostatic cancer	let-7i, miR-16, miR-24, miR-26a, miR-26b, miR-34b, miR-92b, miR-93, miR-103, miR-106a, miR-141, miR-195, miR-197, miR-223, miR-298, miR-328, miR-346, miR-375, miR-1290 (S) [84,85] let-7e, let-7c, miR-20a, miR-21, miR-30c, miR-130b, miR-145, miR-1811a-2 *, miR-221, miR-301a, miR-326, miR-331-3p, miR-432, miR-574-3p, miR-622, miR-625 *, miR-1285, miR-2110, miR-141, miR-1290 (PE) [25,86,99] miR-107, miR-574-3p, miR-141-5p, miR-21-5p, miR-34a, miR-483-5p (PE) [87] miR-375, miR-21, let-7c (UE) [95] miR-196a-5p, miR-501-3p (UE) [96] miR-103a-3p, let-7a-5p (P) [89] let-7a (B) [66] miR-20a (P) [66] miR-30a (P) [66] miR-30c (P) [66] miR-375 (P) [66] miR-34b, miR-16 (S) [66] miR-141 (S, PV, SE) [66]	piR-016658, piR-020496 (PV) [26]	
**Gynaecologic**	Breast cancer	miR-195, let-7, miR-155, miR-138 (B); miR-214, miR-15a, miR-18a, miR-107, miR-425, miR-133a, miR-139-5p, miR-143, miR-145, miR-365, miR-484, miR-1246, miR-1307-3p, miR-6861-5p, miR-4634, miR-6875-5p, miR-155, miR-19a, miR-181b, miR-24, miR-1, miR-92a, miR-133a, miR-133b, let-7c, miR-182, miR-155, miR-21, miR-126, miR-155, miR-199a, miR-335, miR-201, let-7b, miR-4270, miR-1225-5p, miR-188-5p, miR-1202, miR-148b-3p, miR-652-3p, miR-10b-5p, miR-18b, miR-103, miR-107, miR-652, miR-10b, miR-34a, miR-155, miR-29b-2, miR-155, miR-197, miR-205, miR-92a, miR-21, miR-21-5p, miR-375, miR-205-5p, miR-194-5p, miR-382-5p, miR-376c-3p, miR-411-5p, miR-34a, miR-93, miR-373, miR-17, miR-155, miR-125b, miR-122, miR-375, miR-155 (S); miR-127-3p, miR-376a, miR-148b, miR-409-3p, miR-652, miR-801, miR-148b, miR-133a, miR-409-3p, miR-505-5p, miR-125b-5p, miR-21-5p, miR-96-5p, miR-4281, miR-1207-5p, miR-642b-3p, miR-1290, miR-3141, miR-10b, miR-373 (P)[90] miR-101, miR-372, miR-373 (SE) [67] miR-10, miR-16, miR-18a, miR-19a, miR-29a, miR-34a, miR-34b/c, miR-125a, miR-375 (S) [66] miR-106b, miR-125b (P) [66] miR-34a (B) [66] miR-155 (S, S/P) [66]		RNU6 (S) [174]
	Ovarian cancer	miR-214, miR-140, miR-147, miR-135b, miR-205, miR-150, miR-149, miR-370, miR-206, miR-197, miR-634, miR-485-5p, miR-612, miR-608, miR-202, miR-373, miR-324-3p, miR-103, miR-593, miR-574, miR-483, miR-527, miR-603, miR-649, miR-18a, miR-595, miR-193b, miR-642, miR-557, miR-801, let-7e (SE) [100] miR-193a-5p, miR-21, miR-375, miR-210, miR-150-5p, miR-181-5p, miR-142-3p, miR-26a-5p, let-7d-5p, miR-374a-5p, miR-766-3p, miR-200a-3p, miR-130b-3p, miR-328-3p, miR-1246, miR-595, miR-2278, miR-376a, miR-125b, miR-199a, miR-7, miR-429, miR-25, miR-93, miR-200a, miR-200b, miR-200c, miR-145, miR-200c, miR-141, let-7i-5p, miR-122, miR-152-5p, miR-25-3p, miR-22, miR-93, miR-451, miR-106b, miR-21, miR-92, miR-221 (S) [92] miR-373, miR-200a, miR-200b, miR-200c, miR-21, miR-92, miR-155, miR-127, miR-93, miR-99b, miR-126, miR-29a, miR-21, miR-141, miR-203, miR-205, miR-214 (SE) [92] miR-148a, miR-200b, miR-26a, miR-625-3p, miR-720, miR-1274a, miR-19b, miR-223, miR-16, miR-150, miR-20a, miR-126, miR-1290, miR-205, let-7f, miR-16, miR-21, miR-191, miR-4284 (P) [92] let-7f (P) [66] let-7i (S/P) [66] let-7b [66] miR-92, miR-106b, miR-29a (S) [66]		RNU2-1f (S) [175]
	Endometrial cancer	miR-9, miR-92a, miR-99a, miR-100, miR-199b, miR-1228 (P) [176] miR-9, miR-1228, miR-9, miR-92a (P) [177] miR-21, miR-222, miR-223, miR-186 and miR-204 (S) [178,179] miR-203 (S) [180] miR-21 (S) [181] miR-887-5p (S) [182] miR-106b (U) [176] miR-200c-3p (U) [183]		
	Cervical cancer	miR-101, miR-127, miR-142-3p, miR-150, miR-205, miR-21, miR-24, miR-425-5p, miR-486-5p, miR-494 (S) [184] miR-21, miR-146a, miR-155, miR-182, miR-200c, let-7b, miR-145 (S) [185] miR-9, miR-10a, miR-20a, miR-196a (S) [186] miR-21, miR-29a, miR-25, miR-200a, miR-486-5p (S) [187] miR-92a (S) [188] miR-646, miR-141-5p, miR-542-3p (S) [189] miR-205 (S) [190]		
**Haematologic**	Hodgkin Lymphoma	miR-144, miR-143, miR-129-5p, miR-182, miR-411, miR-126-3p, miR-433, miR-23a, miR-24, miR-423-5p (B) [191]	piR-651 (S) [192]	
	Non-Hodgkin Lymphoma	miR-92a, miR-638 (P) [128] *DLBCL* miR-155, miR-210 (S); miR-221 (P)[66] miR-15a, miR-16–1, miR-34a, miR-155, miR-29c (S) [128] miR-425, miR-141, miR-197, miR-145, miR-345, miR-200c, miR-324-5p, let-7i-5, miR-424, miR-222 (B), [191] miR-34a-5p, miR-323b-3p, miR-431-5p, miR-125b, miR-130a, miR-155, miR-200c, miR-29c, miR-145, miR-451, miR-17, miR-20b, miR-210, miR-296, miR-15a-3p, miR-21-5p, miR-210-5p, miR-181a-5p, miR-155-5p, miR-210-3p, miR-16-1, miR-29c, miR-155, miR-34a, miR-223, miR-155, miR-210 (S); miR-21 (S,P); miR-155 (EV from serum)[193] *NTCL* miR-221 (P) [128] *PNCSL* miR-21 (S) [128] miR-21, miR-19, and miR-92a (CSF) miR-451, miR-711, miR-935, miR-223, miR-125b [194]		
	Chronic leukemia	*CLL* let-7a, let-7c, let-7f, let-7g, let-7i, miR-10a, miR-15a, miR-19a, miR-20a, miR-21, miR-22, miR-24, miR-26b, miR-29a, miR-29b, miR-29c, miR-30b, miR-31-5p, miR-33, miR-34a, miR-34a-5p, miR-92, miR-95, miR-99a, miR-100, miR-101, miR-106b, miR-107, miR-123, miR-125a, miR-125a, miR-126, miR-126, miR-130a, miR-132, miR-134, miR-136, miR-138, miR-139, miR-140, miR-141, miR-142-5p, miR-143, miR-146a, miR-147a, miR-148a, miR-150, miR-150-5p, miR-151-3p, miR-154, miR-155, miR-155-5p, miR-181a, miR-181b, miR-182, miR-183, miR-184, miR-185, miR-190, miR-191, miR-192, miR-193b, miR-196-2, miR-197, miR-198, miR-199a, miR-206, miR-212, miR-217, miR-220, miR-223, miR-326, miR-323-3p, miR-632, miR-337-3p, miR-338, miR-342-3p, miR-363, miR-367, miR-370, miR-374b, miR-377, miR-424, miR-442, miR-451, miR-453, miR-484, miR-491, miR-494, miR-572, miR-582-5p, miR-636, miR-640, miR-660, miR-923, miR-1201, miR-1202, miR-1203, miR-3676 (B) [130] miR-195, miR-199b, miR-320, miR-432, (P) [130] *CML* miR-17, miR-18a, miR-20a, miR-21, miR-27a, miR-155 (B) [195] miR-122-5p, miR-16-5p, miR-451a and miR-92a-3p, miR-4644, miR-6075, miR-4656, miR-5739 (B) [196] miR-90, miR-150, miR-16, miR-17, miR-20, miR-21, miR-29, miR-92a, miR-101, miR-106, miR-126, miR-146, miR-150, miR-152, miR-155, miR-451, miR-568, miR-607 (B) [197]		
	Acute leukemia	*AML* miR-92a, miR-143, miR-342, miR-100, miR-196a, miR-146a, miR-29a, miR-142-3p, miR-29a, miR-142-3p, miR-150, miR-342, miR-150, miR-342 (P) [128] miR-21, miR-155, miR-210, miR-221, miR-10a-5p, miR-93-5p, miR-129-5p, miR-155-5p, miR-181b-5p, miR-320d (S) [128] *ALL* miR-155 (S, P) [66] miR-128b, miR-204, miR-218, miR-331, miR-135b, miR-132, miR-199, miR-139, miR-150, miR-519e; miR-487b; miR-361; miR-142-3p; miR-222; miR-339, miR-451; miR-373; miR-296; miR-485-3p; miR-483, miR-196a, miR-383, miR-542-5p, miR-133a (B), miR-708, miR-511, miR-708, let-7b, miR-708, miR-210, miR-181b, miR-345, miR-324-5p, and miR-125b, miR-23a, miR-27a, miR-23b, miR-24 (B) [198]		
	Multiple myeloma	miR-19a (S)[66] miR-92a, miR-483-5p, miR-483-5p, miR-20a (P) [128] miR-29a, miR-34a, let-7e, miR-19a, miR-92a, miR-202, miR-720, miR-1308 (S) [128]		
**Central Nervous System (CNS)**	Glioma	miR-21 (B, P, S) miR-203, miR-137, miR-185, miR-210, miR-205, miR-221/222, miR-29, miR-397a/b/c, miR-125b, miR-497, miR-182, miR-128, miR-451a, miR-15b-5p, miR-23a, miR-133, miR-150 *, miR-197, miR-497, miR-548b-5p, miR-15b-5p, miR-16-5p, miR-19a-3p, miR-19b-3p, miR-20a-5p, miR-106a-5p, miR-130a-3p, miR-181b-5p, miR-208a-3p, miR-17, miR-130a, miR-10b, miR-93, miR-590-3p, miR-454 (S) miR-122, miR-128, miR-342-3p, miR-454-3p (P) miR-15b, miR-16, miR-128, miR-342-3p (B) miR-222, miR-124-3p, miR-301a, miR-320, miR-574-3p (SE) miR-21, miR-10b; miR-200, miR-21, miR-21, miR-15b, miR-451, miR-711, miR-935, miR-223, miR-125b, miR-21-5p, miR-218-5p, miR-193b-3p, miR-331-3p, miR-374a-5p, miR-548c-3p, miR-520f-3p, miR-27b-3p, miR-130b-3p (CSF) [91]		
	Medulloblastoma	*Diagnostic flowchart to distinguish Medulloblastoma from other CNS cancers:* miR-451, miR-711, miR-935, miR-125b, miR-223 (CSF) [194]		
	Retinoblastoma	miR-338-5p (S) [199] miR-18a (S) (S) [200] miR-17, miR-18a, miR-20a (S) [201] miR-320, miR-let-7e, miR-21(S) [202]		
**Endocrine**	Thyroid cancer	miR-25-3p, miR-451a (P) [203] miR-346, miR-10a-5p, miR-34a-5p (P) [204] miR-124-3p and miR-9-3p (P) [205] miR-146b, miR-155 (P) [206] miR-222, miR146b (P) [207] miR-144, miR-34a (P) [208] miR-222, miR-221, miR-146b, miR-21 (S) [209] miR-95, miR-190 (S) [210] miR-222, miR-221 miR-146b (S) [211] miR-30a-5p (S) [212] miR-146a-5p, miR-199b-3p, let7b-5p, miR-10a-5p (S) [213] miR-95, miR-190 (S) [214] miR-222 (S) [215] let-7e, miR-151-5p, miR-222 (S) [216] miR-375, miR-34a, miR-145b, miR-221, miR-222, miR-155, Let-7, miR-181b (S) [217] miR-146b-5p, miR-222-3p, miR-155-5p, and miR-378a-3p (B) [218] miR-21, miR-31, miR-181a-5p (PE) [218]		
**Bone**	Osteosarcoma	miR-487a, miR-493-5p, miR-501-3p and miR-502-5p (S) [219] miR-25-3p (S) [220] miR-215-5p, miR-642a-5p (S) [221] miR-101 (S) [222] miR-124 (S) [223] miR-375 (S) [224] miR-598 (S) [225] miR-95-3p (S) [226] miR-300 (S) [227] miR-497 (S) [228] miR-326 (S) [229] miRNA-223 (S) [230] miR-421 (S) [231] miR-106a-5p, miR16-5p, miR-20a-5p, miR-425-5p, miR451a, miR-25-3p, miR139-5p(S) [232] miR-152 (S) [233] miR-191 (S) [234] miR-221 (S) [235] miR-199a-5p (S) [236] miR-27a (S) [237] miR-195 (S) [238] miR-133b, miR-206 (S) [239] miR-29a, miR-29b, miR-29c (S) [240] miR-663a (P) [241] miR-195-5p, miR-199a-3p, miR-320a, miR-374a-5p (P) [242] miR-139-5p (P) [243] miR-Let7a (B) [244] miR-148a (B) [245]		
**Skin Cancers**	Melanoma	miR-1260a, miR-126-5p, miR-1280, miR-145-5p, miR-150-5p, miR-155-5p, miR-16-5p, miR-182-5p, miR-193b-3p, miR-197-3p, miR-200c-3p, miR-204-5p, miR-205-5p, miR-206, miR-211-5p, miR-211-5p, miR-221-3p, miR-22-3p, miR-27a-3p, miR-28-5p, miR-301a-3p, miR-30b-5p, miR-373-5p, miR-374-5p, miR-432-3p, miR-4487, miR-451a, miR-4706, miR-4731, miR-509-3p, miR-509-5p, miR-550a-3p, miR-627-5p, miR-629-5p, miR629-5p, miR-720, miR-9-5p (S) [246] miR-126-5p, miR-211-5p, miR-206, miR-16 miR-211, miR-182-5p, miR-301a-3p, miR-193b-3p, miR-197-3p, miR-200c-3p, miR-204-5p, miR-28-5p, miR-27a-3p, miR-29c-5p, miR-324-3p, miR-374a-5p, miR-4487, miR-4706, miR-4731, miR-509-3p, miR-509-5p, miR-550a-3p, miR-627-5p, miR-629-5p, miR-720, miR-205-5p (S) [247] miR-182-5p, miR-193b-3p, miR-200c-3p, miR-204-5p, miR-205-5p, miR-211-5p, miR-301a-3p, miR-720 (S) [248] miR-150-5p, miR-146a, miR-149-3p, miR-155, miR-15b-5p, miR-181a, miR-193a-3p, miR-20a, miR-223, miR-524-5p, miR-125b (P) [247] miR-1258, miR-1264, miR-1269a, miR-1302, miR-1306-5p, miR-138-5p, miR-152-3p, miR-1537-3p, miR-154-5p, miR-1-5p, miR-181b-5p, miR-1910-5p, miR-1973, miR-205-5p, miR-219a-2-3p, miR-2682-5p, miR-27a-3p, miR-299-3p, miR-301a-3p, miR-3131, miR-337-5p, miR-34a-5p, miR-3928-3p, miR-424-5p, miR-431-5p, miR-450a-5p, miR-4532, miR-454-3p, miR-4787-3p, miR-497-5p, miR-520d-3p, miR-522-3p, miR-548a-5p, miR-548ad-3p, miR-548l, miR-553, miR-624-3p, miR-764 (P) [249] miR-134-5p, miR-320a-3p (P) [250] miR-21-5p (P) [246] miR-200c-3p (P, S) [246] miR-125b (SE) [247]		

Abbreviations: SA = Saliva; P = Plasma, SE = Serum exosomes; S = Serum; PV = Plasma vesicles; B = Blood; P = Plasma exosomes; U = Urine; UE = Urine exosomes; SP = Sputum, CSF = Cerebrospinal fluid; BC = Blood cells; F = Feces; BI = Bile; GJ = Gastric Juice; TEP = Tumor educated platelets; CRCR = Colorectal cancer; DLBCL = Diffuse large B-cell lymphoma; NTCL = Extranodal NK/T-cell lymphoma; PNCSL = Primary Central Nervous System Lymphoma; CLL = Chronic lymphocytic leukemia; CML = Chronic myeloid leukemia; AML = Acute myeloid leukemia, ALL = Acute myeloid leukemia, CNS = Central nervous system.

**Table 2:** Long noncoding RNAs and circular RNAs with possible application as diagnostic biomarkers in body fluids.

Cat.	Cancer Type	lncRNA	circRNA
**Digestive **	Oral cancer	*AC007271.3* (S) [307] *HOTAIR* (SA) [300]	*hsa_circ_0001874* (SA) [308] *hsa_circ_0001971* (SA) [308] *circMAN1A2* (S) [309]
	Nasopharyngeal cancer	*MALAT1, AFAP1-AS1, AL359062* (S) [310]	*circRNA_0000285* (S) [311] *circMAN1A2* (S) [309]
	Esophageal cancer	*POU3F3; HNF1A-AS1; SPRY4-IT1* (P) [282] *MIR31HG* (P), [312] *HOTAIR* (S) [313] *POU3F3, SCCA* (P) [282]	*-*
	Hepatocellular carcinoma	*HULC* (P) [281] *RP11-160H22.5, XLOC_014172, LOC149086* (P) [284] *SNHG18* (P) [286] *SNHG1* (P) [290] *GAS5-AS1* (P) [314] *ZFAS1 (P)* [315] *DANCR* (P) [316] *HULC, Linc00152* (P) [317] *SPRY4-IT1* (P) [318] *NEAT* (S) [319] *LRB1* (S) [320] *UCA1* (S) [321] *X91348* (S) [322] *PVT1, uc002mbe.2* (S) [323] *uc001ncr, AX800134* (S) [324] *UCA1, WRAP53* (S) [325] *LINC00161* (S, SE, U) [326] *ENSG00000258332.1, LINC00635* (SE) [327]	*hsa_circ_0001445* (P) [328] *circSMARCA5* (P) [329]
	Cholangiocarcinoma	*PCAT1, MALAT1, CPS1-IT1* (P) [330] *ENST00000588480.1, ENST00000517758.1* (BI) [302]	*-*
	Gallbladder carcinoma	*-*	*-*
	Pancreatic cancer	*ABHD11-AS1, LINC00176, SNHG11* (P) [331] *HOTAIR, PVT1* (SA) [301] *HOTTIP-005, RP11-567G11.1* (P/S) [332]	*circLDLRAD3* (P) [333]
	Gastric cancer	*PANDAR, FOXD2-AS1, SMARCC2* (P) [334] *FAM49B-AS, GUSBP11, CTDHUT* (P) [335] *H19, MEG3* (P) [336] *GACAT2* (P) [337] *HOTAIR* (P) [338] *AK001058, INHBA-AS1, MIR4435-2HG, CEBPA-AS1, CTC-501O10.1, AC100830.4, RP11-210K20.5* (P) [339] *ZFAS1* (P) [280] *UCA1* (P) [340] *TINCR, CCAT2, AOC4P, BANCR, LINC00857* (P) [341] *HULC (S)* [342] *HOXA11-AS* (S) [343] *H19* (S) [344,345] *CTC-497E21.4* (S) [346] *LINC00978* (S) [347] *XIST, BCYRN1, RRP1B, TDRG1* (S) [348] *H19* (S) [349] *CUDR; LSINCT-1; PTENP1* (S) [291] *LINC00152* (P, E) [278] *UEGC1* (PE) [350] *LINC00152, AA174084, UCA1, RMRP, ABHD11-AS1, LINC00982, H19* [351] (GJ) *AA174084* (GJ) [303]	*hsa_circ_0001649* (P) [352] *hsa_circ_0001017, hsa_circ_0061276* (P) [353] *hsa_circ_0000745* (P) [354] *hsa_circ_0000520* (P) [355] *circ-KIAA1244* (P) [356] *hsa_circ_0000190* (P) [357] *hsa_circ_0000467* (P) [358] *hsa_circ_0000181* (P) [359]
	Colorectal cancer (CRC)	*ATB, CCAT1* (P) [360] *91H, PVT-1, MEG3* (P) [361] *CCAT2, HULC* (P) [362] *BLACAT1* (P) [363] *B3GALT5-AS1* (P) [364] *CCAT2* (S) [293] *LOC285194, RP11-462C24.1, Nbla12061* (S) [365] *NR_029373, NR_034119* (S) [366] *CCAT1, HOTAIR* (S) [367] *CRNDE-p* (SE) [158] *CRNDE-h* (SE) [294] *CCAT2* (SE) [293] *LNCV6_116109, LNCV6_98390, LNCV6_38772, LNCV_108266, LNCV6_84003, LNCV6_98602* (PE) [368] *CRNDE-h* (SE) [294] *BCAR4 (+mRNA KRTAP5-4 and MAGEA3)* (SEV) [369]	*hsa_circ_0001649* (S) [370] *circVAPA (hsa_circ_0006990)* (S) [371]
**Respiratory**	NSCLC	*GAS5, SOX2OT* (P) [288] *H19* (P) [372] *HOTAIR* (P) [373] *Linc00152* (P) [374] *SNHG1, RMRP* (P) [375] *GAS5* (P) [376] *SPRY4-IT1, ANRIL, NEAT1* (P) [377] *RP11-397D12.4, AC007403.1, ERICH1-AS1* (P) [283] *UCA1* (P) [378] *RP11-397D12.4, AC007403.1, ERICH1-AS1* (P) [283] *TUG1* (S) [379] *PCAT6* (S) [380] *XIST, HIF1A-AS1* (S) [381] *AFAP1-AS1* (S) [382] *SOX2OT, ANRIL, CEA, CYFRA21-1, SCCA* (S) [383] *XLOC_009167* (B) [384] *MALAT1* (SE) [292] *GAS5* (SE) [385] *SOX2-OT* (PE) [386] *SLC9A3-AS1, PCAT6* (EV from P) [387]	*-*
	Laryngeal cancer	*HOTAIR* (S) [388]	*-*
**Urologic **	Renal cell carcinoma	*lncARSR* (P) [285] *LET, PVT1, PANDAR, PTENP1, linc00963* (S) [389]	*-*
	Bladder cancer	*TUC338* (P) [390] *H19* (SE) [391] *PCAT-1, UBC1, SNHG16* (SE) [392] *H19 (U)* [393] *miR-497-HG (together with miR-324-5p, miR-4738-3p and RCAN1 FOSB mRNA) (U)* [173] *uc004cox.4, GAS5* (U) [394] *HOTAIR, HOX-AS2, ANRIL, HYMA1, LINC00477, LOC100506688, OTX2-AS1* (UE) [295] *UCA1-201, UCA1-203, MALAT1, LINC00355* (UE) [395] *MALAT1, PCAT-1, SPRY4-IT1* (UE) [396] *MEG3, SNHG16, MALAT1* (S) [397]	*circPRMT5* (SE, UE) [398]
	Prostatic cancer	*TUC338* (P) [399] *BRE-AS1* (P) [400] *MALAT1* (P/S) [401] *PCA3/PSA* (U) [402] *PRCAT17.3, PRCAT38* (U) [403] *PCA3, ERG* (UE) [296,297,299] *TP53COR1* (UE) [298] *FR0348383* (U) [404]	*-*
**Gynaecologic**	Breast cancer	*ANRIL, HIF1A-AS2, UCA1* (P) [287] *H19* (P) [405] *MALAT1* (S) [406] *RP11-445H22.4* (S) [407]	*hsa_circ_0001785* (P) [408]
	Ovarian cancer	*-*	*circMAN1A2* (S) [309]
	Endometrial cancer	*-*	*hsa_circ_0109046* (SEV) [409] *hsa_circ_0002577* (SEV) [409]
	Cervical cancer	*FALEC* (P) [410] *HOTAIR, PVT1, XLOC_000303, AL592284.1* (P) [411] *GIHCG* (S) [412] *PVT1* (S) [413]	*-*
**Haematologic**	Hodgkin Lymphoma	*-*	*-*
	Non-Hodgkin Lymphoma	*-*	*-*
	Chronic leukemia	*LincRNA-p21* (P) [289]	*-*
	Acute leukemia	*-*	*-*
	Multiple myeloma	*TUG1; MALAT1; HOTAIR; GAS5* (P) [289] *PCAT-1* (S) [414] *H19* (S) [415]	*-*
**Central Nervous System (CNS)**	Glioma	*miR210HG* (S) [416]	*-*
	Medulloblastoma	*-*	*-*
	Retinoblastoma	*-*	*-*
**Endocrine**	Thyroid cancer	*GAS8-AS1* (P) [417]	*circMAN1A2* (S) [309] *hsacirc_007293, hsacirc_031752, hsacirc_020135* (SE) [418]
**Bone**	Osteosarcoma	*UCA1* (S) [393]	*hsa_circ_0000885* (S) [419]
**Skin Cancers**	Melanoma	*PVT1* (S) [420]	*-*

Abbreviations: SA = Saliva; P = Plasma, SE = Serum exosomes; S = Serum; PV = Plasma vesicles; B = Blood; P = Plasma exosomes; U = Urine; UE = Urine exosomes; SP = Sputum, CSF = Cerebrospinal fluid; BC = Blood cells; F = Feces; BI = Bile; GJ = Gastric Juice; TEP = Tumor educated platelets; CRC = Colorectal cancer; DLBCL = Diffuse large B-cell lymphoma; NTCL = Extranodal NK/T-cell lymphoma; PNCSL = Primary Central Nervous System Lymphoma; CLL = Chronic lymphocytic leukemia; CML = Chronic myeloid leukemia; AML = Acute myeloid leukemia, ALL = Acute myeloid leukemia.

## Supplementary Materials

The following data are available online at https://www.mdpi.com/2072-6694/11/8/1170/s1, Table S1: (A) Comparison of miRNA-cancer associations in tissues and body fluids: T is associated only in tissues, B only in body fluids and BT in both; (B) Number of microRNAs associated to each cancer type in body fluids and/or tissues; (C) Number of cancer types associated to each microRNA in body fluids and/or tissues.
